# The Plant NF-Y DNA Matrix *In Vitro* and *In Vivo*

**DOI:** 10.3390/plants8100406

**Published:** 2019-10-10

**Authors:** Nerina Gnesutta, Matteo Chiara, Andrea Bernardini, Matteo Balestra, David S. Horner, Roberto Mantovani

**Affiliations:** Dipartimento di Bioscienze, Università degli Studi di Milano, Via Celoria 26, 20133 Milano, Italy; nerina.gnesutta@unimi.it (N.G.); matteo.chiara@unimi.it (M.C.); andrea.bernardini@unimi.it (A.B.); matteo.balestra@studenti.unimi.it (M.B.); david.horner@unimi.it (D.S.H.)

**Keywords:** Transcription Factor, plant NF-Y, TFBS, DNA matrix, LEC1

## Abstract

Nuclear Factor Y (NF-Y) is an evolutionarily conserved trimer formed by a Histone-Fold Domain (HFD) heterodimeric module shared by core histones, and the sequence-specific NF-YA subunit. In plants, the genes encoding each of the three subunits have expanded in number, giving rise to hundreds of potential trimers. While in mammals NF-Y binds a well-characterized motif, with a defined matrix centered on the *CCAAT* box, the specificity of the plant trimers has yet to be determined. Here we report that *Arabidopsis thaliana* NF-Y trimeric complexes, containing two different NF-YA subunits, bind DNA *in vitro* with similar affinities. We assayed precisely sequence-specificity by saturation mutagenesis, and analyzed genomic DNA sites bound *in vivo* by selected HFDs. The plant NF-Y *CCAAT* matrix is different in nucleotides flanking *CCAAT* with respect to the mammalian matrix, *in vitro* and *in vivo*. Our data point to flexible DNA-binding rules by plant NF-Ys, serving the scope of adapting to a diverse audience of genomic motifs.

## 1. Introduction

In eukaryotes, the access of enzymes that transcribe, replicate, repair and recombine DNA is regulated by chromatin, whose fundamental unit is the nucleosome. Protein complexes with enzymatic activities modify histones in nucleosomes through post-translational modifications, as well as DNA itself [1]. The recruitment of these machineries to the appropriate genomic locations is driven by transcription factors (TFs) bound to specific sequences in promoters and enhancers. As for RNA production, selective and often synergistic binding of TFs to their DNA *cis*-elements governs transcription initiation [2]. Furthermore, a subset of TFs is structurally built to penetrate “closed” genomic areas, and initiates the process of chromatin opening by recruiting other TFs, modifying machines and the general apparatus required for activation and elongation of transcription.

Nuclear Factor Y (NF-Y) is widely considered a pioneer TF in mammals, as well as plants [3,4,5,6,7]. It is formed by the NF-YA, NF-YB and NF-YC subunits. NF-YB and NF-YC have a histone fold domain—HFD—which mediates their heterodimerization *à la* H2A/H2B core histones, forming a platform for NF-YA association [8]. In mammals, the DNA sequence recognized is the *CCAAT* pentanucleotide, a box commonly found in promoters, as well as distal regulatory elements [9]. The 3D structures of the fungi and mammals hetero-trimers are known, and the details of DNA interactions well understood: The HFD dimer forms a non-sequence-specific surface, while *CCAAT* box recognition is mediated by specific contacts of NF-YA [10,11]. The latter initiates DNA bending for the HFD dimer to provide wide surfaces engaging with DNA on both sides of *CCAAT*: Overall, 25/30 nucleotides are contacted by the trimer. 

NF-Y genes are extremely conserved, in all eukaryotes. Unique to plants, all three NF-Y genes have undergone multiple duplications, resulting in considerably expanded gene families [12]: Seven to fifteen members for each subunit, depending on the species. Phenotypic analysis of NF-Y genes mutant plants suggests that they are involved in numerous key processes, from embryo development, flowering, roots formation, to responses to adverse conditions [13,14,15]. The second plant-specific feature is the presence of a second group of NF-YA-like TFs: CCT (CONSTANS (CO), CONSTANS-LIKE, TIMING OF CAB EXPRESSION 1 (TOC1)) proteins share the domain required for HFD interactions and DNA-binding [16]. Indeed, we—and others—went on showing that Arabidopsis CO and the related rice HEADING DATE 1 (Hd1) form a stable hetero-trimer, NF-CO, with NF-YB/NF-YCs [17,18,19]. Because of differences in amino acid composition within the CO DNA-binding subdomain, NF-CO binds an element different from *CCAAT*, termed CORE (CO-Responsive Element), which we characterized by saturation mutagenesis [18]. 

The binding of mammalian NF-Y to *CCAAT* was functionally dissected in numerous mutagenesis studies [9]; binding *in vivo* was assessed in genomic studies, including by the vast collection of transcription factor binding affinity profiles compiled by the ENCODE consortium [3,20,21,22]. It is quite clear that each nucleotide of the *CCAAT* box is mandatory for the association, but flanking nucleotides are also extremely important: Overall, a matrix of 10 bp, as originally proposed, turned out to be the target of NF-Y *in vivo* ([9], and all references therein). 

On the contrary, the sequence preference of plant NF-Ys has yet to be determined. Based on sequence identity in conserved domains of the three subunits, and on our initial set of *in vitro* experiments, it is largely assumed that the matrix is centered on a *CCAAT* motif and identical to the mammalian one. This assumption needs to be verified, for the following reasons: (i) Inspection of Arabidopsis promoters for *CCAAT* found a modest enrichment of the pentanucleotide, but not of flanking nucleotides [23]. (ii) The only functionally important *CCAAT* sequence known in plants, to which NF-Ys bind to *in vivo*, lies within the −5.3 kb enhancer of the Arabidopsis *FT* gene [24,25,26]: It is far from an optimal *CCAAT*, according to mammalian standards. (iii) Bioinformatic analysis of dys-regulated genes in double *nf-yb2 nf-yb3* and triple *nf-yc3 nf-yc4 nf-yc9* plants found significant enrichment of *CORE*s, but not *CCAAT* elements in promoters of down-regulated genes [18]: This even raised a question on whether the major purpose of HFDs could be to form CORE-binding NF-CO, rather than NF-Y. 

To thoroughly address this issue, we present systematic analyses of the *in vitro* DNA-binding specificity of two AtNF-Y trimers, as well as targets selection of HFD-complexes *in vivo*.

## 2. Results

### 2.1. DNA Sequence-Specificity of the NF-Y Trimer

**Figure 1:**
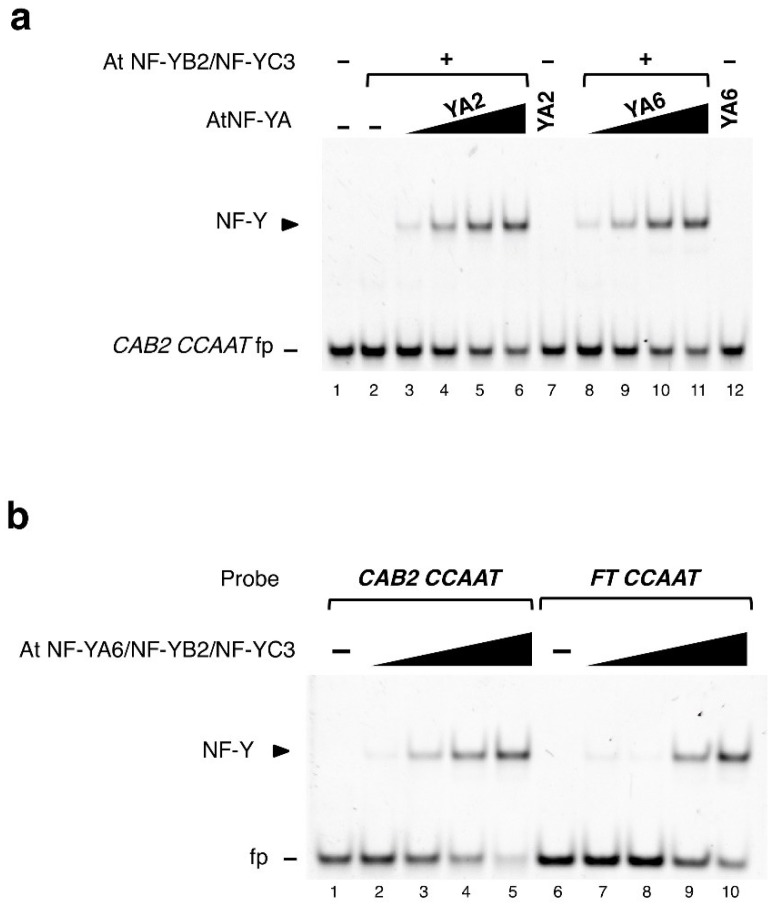
AtNF-YAs functionally trimerize with At NF-YB2/NF-YC3 in electrophoretic mobility shift assay (EMSA). EMSAs were performed to assess AtNF-Y trimerization and DNA binding: (**a**) At NF-YA2 or NF-YA6 subunits were incubated at increasing concentrations (60, 120, 180, 240 nM) with the *CAB2 CCAAT* probe (20 nM) in presence of the At NF-YB2/NF-YC3 histone fold domain (HFD) dimer (30 nM). As negative controls, AtNF-YAs (lanes 7, 12; 240 nM) or the HFD dimer (lane 2; 30 nM) were incubated alone with the probe; (**b**) AtNF-Y trimer composed of At NF-YB2/NF-YC3 HFD dimer and AtNF-YA6 (1:4.5 fixed molar ratio) was used in binding reactions at increasing concentrations (40, 50, 60, 70 nM) with *CAB2* promoter (−65 bp) or *FT* enhancer (−5.3 kb) *CCAAT* oligonucleotide probes (20 nM). Lanes 1, 6: Probe alone. Below, the 31mers *CCAAT* oligonucleotides’ sequences used in EMSA. The *CCAAT* pentamer is highlighted in boldface. Underlined bases were mutagenized in competitor oligos used in EMSA competition analyses shown in Figure 2a,b. (**a**,**b**) On the left side of each gel, an arrowhead indicates the AtNF-Y/DNA complex; fp: Free probe; purified recombinant proteins are shown in Figure S1.

The DNA sequence-specificity of mammalian NF-Y has been precisely assessed *in vitro* by saturation mutagenesis and SELEX studies (reviewed in Reference [9]), and later confirmed by ChIP-Seq experiments [3,22]. On the other hand, the selectivity of plant trimers has never been tested. The only *CCAAT* box shown to be functional with genetic experiments is the Arabidopsis *FT* −5.3 Kb enhancer: We previously showed that this *CCAAT* could bind to NF-Y trimers [18,25,26]. To identify functional elements located in proximal promoters potentially bound by NF-Y, we surveyed the literature: Interestingly, we noticed that *Lhcb* (light-harvesting clorophyll a/b-binding protein) genes from different plant species display a conserved 10-bp sequence which includes a perfect *CCAAT* [27,28,29]. Among these, the Arabidopsis *CHLOROPHYLL A/B BINDING PROTEIN 2* (*CAB2*; lhcb1*1) −111 to −38 promoter region, was shown capable of interacting with a protein of nuclear extracts [30]. To evaluate AtNF-Y binding of the *CAB2* (−65) *CCAAT* box, we performed electrophoretic mobility shift assays (EMSAs) with the purified recombinant trimer composed of the At NF-YB2/NF-YC3 HFD subunits, and either AtNF-YA6 or AtNF-YA2. These NF-YA subunits were selected based on their relative divergence [12] and because we previously characterized *in vitro* binding to the *FT* −5.3 kb *CCAAT* element [18,26]. Indeed, dose-response EMSAs show binding to the Cy5-labeled *CCAAT* 31 bp *CAB2* oligonucleotide, confirming that the two AtNF-Y trimers form complexes with similar efficiency (Figure 1a). Next, we assessed the binding affinity of the AtNF-YA6 trimer for the *CAB2 CCAAT* element in parallel with the *FT CCAAT*, as shown in Figure 1b, the *FT CCAAT* probe also formed a complex, as expected, but with reduced affinity.

**Figure 2:**
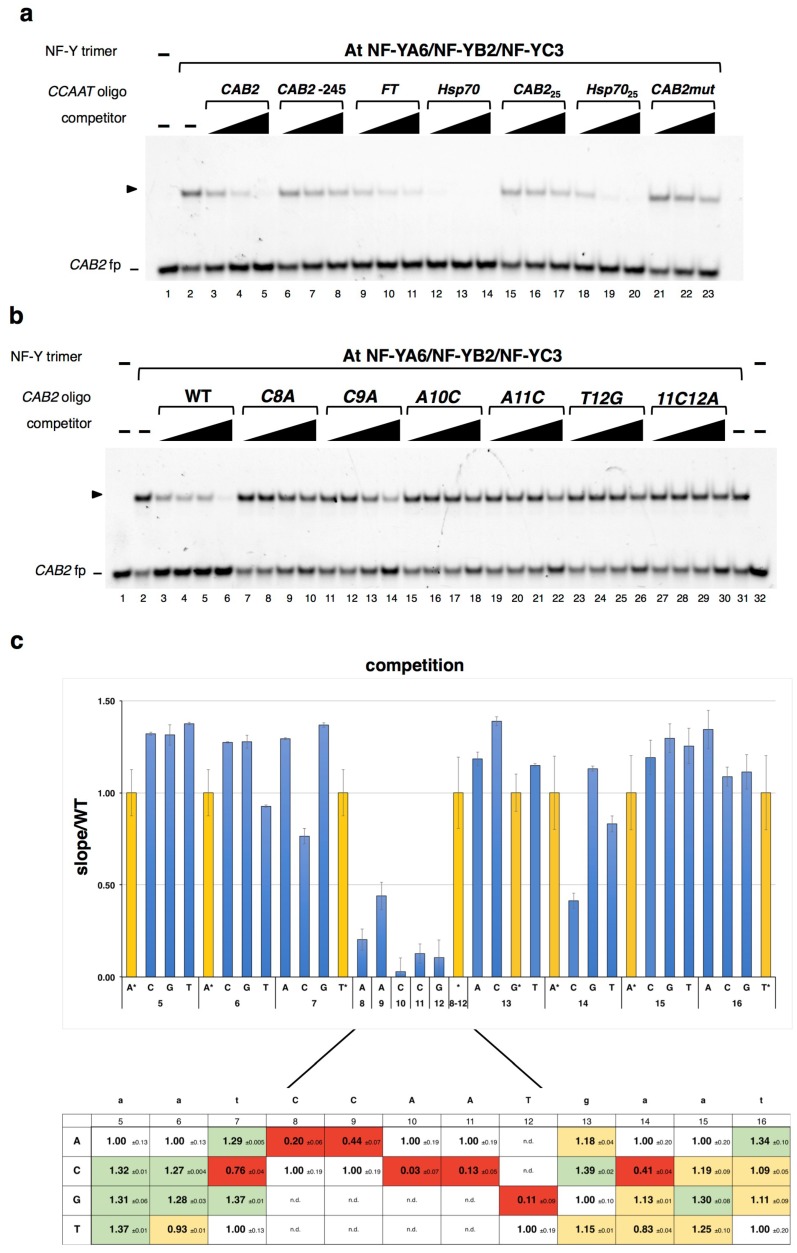
AtNF-Y binds *CCAAT* DNA with sequence-specificity. (**a**) EMSA competition analysis of AtNF-Y *CCAAT* box specificity. The At NF-YA6/NF-YB2/NF-YC3 trimer (30 nM) was incubated with the *CAB2 CCAAT* (−65) (*CAB2*) probe (20 nM; lanes 2−23), with the addition of the indicated unlabeled competitor 31mer oligos at increasing concentrations (1X, 5X, 25X fold excess), or TE buffer alone (-) (lane 2). Lane 1: Probe alone. *CAB2mut*: *CAB2* (−65) *C8A* mutant oligo. *Hsp70_2_*_5_, *CAB2_25_*: 25 bp oligos. (**b**) *CAB2 CCAAT* 31mer competitor oligos containing single bp transversion mutations of each *CCAAT* bp of the *CAB2* sequence (positions 8−12), or two bp mutant (*11C12A*) were used in competition EMSAs of At NF-YA6/NF-YB2/NF-YC3 trimer, as indicated above, at increasing concentrations (1X, 2X, 5X, 25X fold excess). The wild-type *CAB2* unlabeled oligo (WT) was used as a positive control. Lanes 1, 32: Probe alone. Lanes 2, 31: AtNF-Y binding reactions incubated with buffer alone. In (**a**) and (**b**) representative gels of competition experiments are shown. An arrowhead indicates the AtNF-Y/DNA complex. fp: Free probe. (**c**) Quantification of saturation mutagenesis off-rate EMSAs. *CAB2 CCAAT* mutant oligonucleotides spanning positions 5−16 (underlined sequence in Figure 1b; bp positions numbered according to the mammalian crystal structure complex *HSP70 CCAAT* nucleotides ([11]; PDB-code: 4AWL) were used in competition EMSA experiments and bound DNA was quantified in each dose curve data point (0, 1X, 5X fold excess). The graph values represent the regression line slope of each oligo *versus* the WT oligo slope (slope/WT). For each position, an asterisk (*) and corresponding yellow shaded bars represent the WT oligo control. The competition values (+/− s.d.) are displayed in the table below, with red, green or yellow shading highlighting the reduced, increased, or similar, respectively, competition rates of each oligo as compared to the WT control. n.d.: Not determined.

Next, we assessed the sequence-specificity of the At NF-YA6/NF-YB2/NF-YC3 trimer for DNA. Considering the higher avidity observed for the *CAB2 CCAAT* site as compared to the *FT* distal element, to obtain maximum sensitivity of the assays, we decided to use the *CAB2* promoter element for measurements of plant NF-Y DNA selectivity. First, the specificity of the *CAB2* complex was verified by challenging with unlabeled *CCAAT* oligos of different lengths and origins: The WT *CAB2* (−65 bp from transcription start site (TSS)), a second *CCAAT* element found in the *CAB2* promoter (−245 bp), the *FT CCAAT* distal element, as well as the high affinity—for mammalian NF-Y—HSP70 *CCAAT* (Oligonucleotide sequences are listed in Table S1). We also tested 25 bp competitors designed on the crystallized human *HSP70 CCAAT* oligo and on the *CAB2 CCAAT*. Figure 2a shows that, bar *CAB2* −245, all compete efficiently, although, as expected from dose response EMSAs, the *FT CCAAT* shows lower competition rates (i.e., less affinity) than the WT *CAB2*; the human *HSP70 CCAAT* competes extremely well. The shorter versions of the *CAB2* −65 and of the *HSP70* oligos compete with decreased efficiency. The competition was substantially decreased by a single mutation in the *CCAAT* pentamer (*CAB2mut*, *ACAAT*, see *C8A* oligo in panel (b)). We further analyzed the specificity of the plant NF-Y trimer—based on AtNF-YA6—for *CCAAT* by challenging it with *CAB2* oligo competitors containing single mutations in the pentanucleotide: Figure 2b,c shows that transversions in any of the five central base-pairs lead to a sharp decrease in affinity. Similar results were obtained with the AtNF-YA2 based trimer (data not shown). In conclusion, the plant NF-Y complexes containing the NF-YA, NF-YB and NF-YC subunits used here are *bona fide CCAAT*-binding entities.

We then challenged the At NF-YA6/NF-YB2/NF-YC3 trimer in off-rates by competition with sets of unlabeled oligos in which *CCAAT*-flanking nucleotides, three at the 5’ and four at the 3’ of the *CAB2 CCAAT,* were individually mutated into the other three nucleotides. The limits of this mutagenesis (see the underlined sequence in Figure 1c) were suggested by our knowledge of different aspects of NF-Y/*CCAAT* contacts, including the 3D structure of the fungi and mammalian complexes. The saturation mutagenesis results are shown in Figure 2c, where nucleotide (*N*) residues of the *CAB2* oligo are numbered according to the *CCAAT* bases in the crystallized human *HSP70* oligo [11]. Increased competition rates of the mutated oligos at position −3 (*N_5_*), indicate that the WT *A* is the least preferred base at this position, with the highest affinity for a *T*. At −2 (*N_6_*), *A* or *T* are equal, while *C* or *G* increase DNA affinity over the WT oligo. At −1 (*N_7_*), purines are preferred over the WT *T*, or *C*, which decreases the affinity. Downstream of *CCAAT*, at +1 (*N_13_*), *C* is preferred over the WT *G*, *A* or *T*. At position +2 (*N_14_*), purines are preferred over pyrimidines, with the *C* mutant oligo showing the lowest affinity. At +3 (*N_15_*) all bases show similar competition rates with the WT *A* being the least preferred base. Finally, at +4 (*N_16_*), a higher affinity for *A* is scored. Overall, we could construct a matrix deriving from these data. 

These data indicate that nucleotides flanking *CCAAT* concur in determining the affinity of the plant trimers to target DNA, showing relevant differences from the mammalian NF-Y matrix (see Section 3).

### 2.2. Analyses of AtNF-YB2, AtNF-YC2 and LEC1 Binding Sites In Vivo

**Figure 3:**
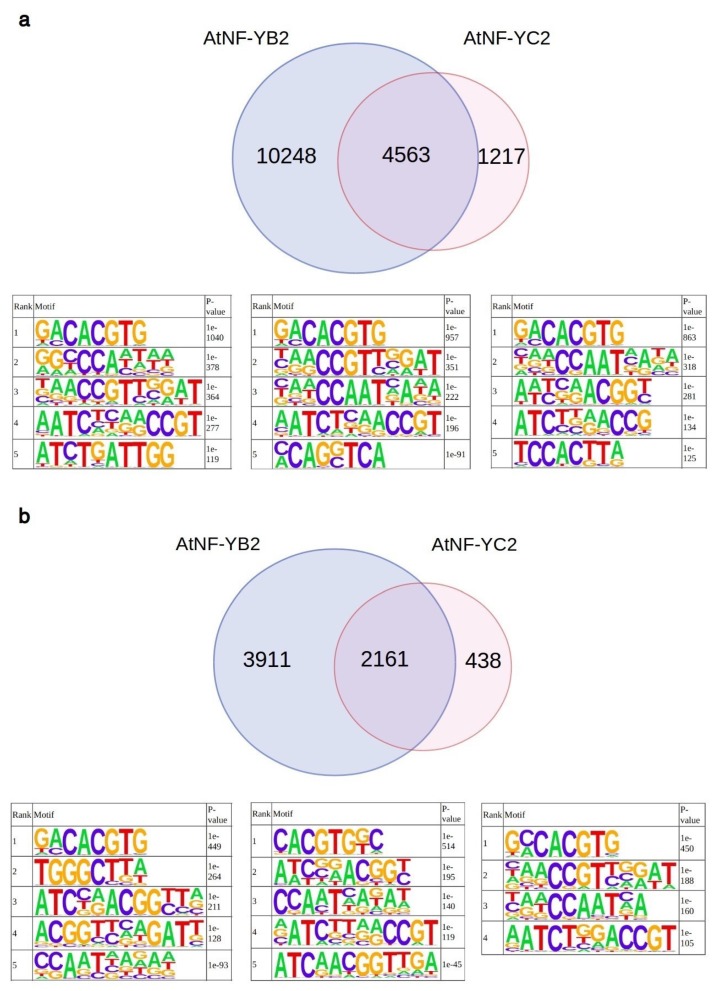
Analysis of AtNF-YB2 and AtNF-YC2 binding sites *in vivo*. Venn diagram displaying the number of peaks detected in the AtNF-YB2, AtNF-YC2 ChIP-Seq experiments and the number of shared peaks [31] with enriched motifs, as recovered by Homer (*p*-value ≤ 10e−30) for AtNF-YB2 (left), AtNF-YC2 peaks (right) and shared peaks (center). (**a**) All Peaks. (**b**) Peaks in promoter regions (defined as genomic intervals from −500 bp to an annotated TSS).

At present, only two published studies incorporate ChIP-Seq data from NF-Y HFD subunits in plants. The first study used epitope-tagged AtNF-YB2 and AtNF-YC2 as a tool for the exploration of transcriptional regulatory circuits underlying ABA responses [31]. To investigate sequence-specificity of NF-Y subunits *in vivo*, we retrieved the original peak calls for NF-Y subunits ChIP-Seq data obtained by this study from (https://www.ncbi.nlm.nih.gov/geo/query/acc.cgi?acc=GSE80568) and performed motif enrichment analysis on 50 base windows centered on the specified peak summits, by means of the HOMER software [32]. Of the 14811 AtNF-YB2, and 5780 AtNF-YC2 peaks identified by Song et al., 4563 (78,9%) were overlapped (Figure 3a). About 40% of the peaks were associated with promoters (<500 nt from annotated TSSs), according to the TAIR10 annotation of the Arabidopsis reference genome. For these peaks overlap between AtNF-YC2 and AtNF-YB2, was even higher, with 2161 of the 2599 promoter peaks (83%) overlapping with AtNF-YB2 (Figure 3b). Significantly enriched (Homer *p*-value lower than 10^−30^) sequence motifs identified by our analyses are displayed in the bottom part of each panel in Figure 3a,b.

Remarkably, all our analyses recovered significant enrichment of *CCAAT*-like motifs, showing a generally purine-rich flanking context, with a notable enrichment—particularly *A*—at positions +2 and +4 (Matrices no. 5, 3, 2 in the left, central, right panels, respectively, in Figure 3a; Matrices no. 5, 3, 3 in the left, central, right panels in Figure 3b). This is consistent with the results of our EMSAs. In accordance with the analyses by Song et al. [31], several other motifs were more frequently observed and more significantly enriched than the canonical *CCAAT* box. In particular, ABRE-like, G box containing elements (*C*[*A/C*]*CACGTG*) and TCP responsive element-like motifs (*GCCCA*). This observation mirrors a previous analysis of promoters of genes differentially expressed in light stressed *co*, *nf-yb2 nf-yb3*, and *nf-yc3 nf-yc4 nf-yc9* mutants, where a *CORE*-like site, as well as a G box variant (*CCACGTG*), but not the *CCAAT* motif were recovered [18].

**Figure 4:**
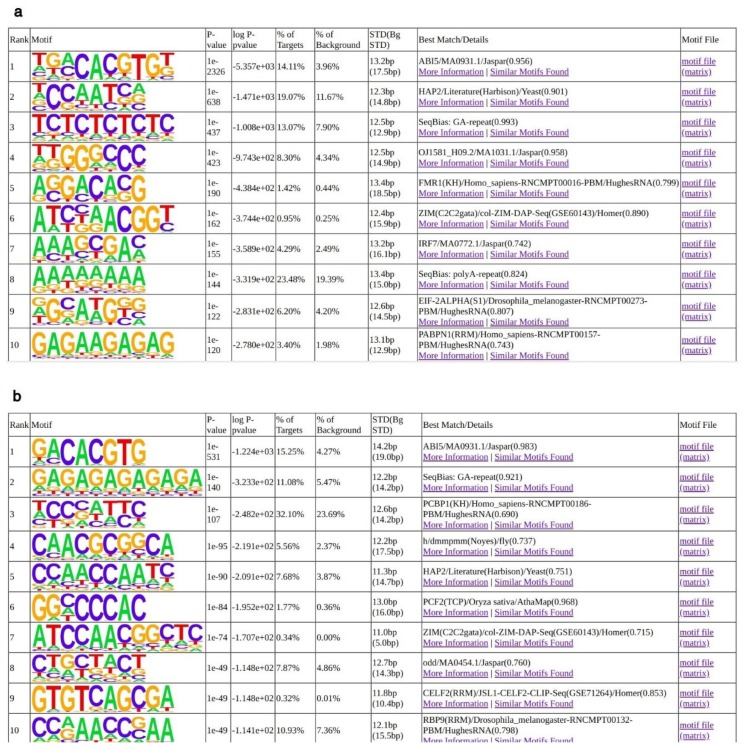
Analysis of LEC1 binding sites *in vivo*. Enriched motifs, as recovered by Homer (*p*-value ≤ 10e−30) for soybean LEC1 peaks [33]. (**a**) All Peaks. (**b**) Peaks in promoter regions (defined as genomic intervals from −500 bp to an annotated TSS).

The second study focused on the characterization of regulatory networks orchestrated by LEC1 (AtNF-YB9) during embryonic development of Arabidopsis and soybean [33]. In this case, we analyzed only the ChIP-Seq data obtained in soybean using a specific anti-LEC1 antibody. In the original work, the authors used a combination of filters, based on expression patterns of genes, in order to identify more than 3400 candidate targets regulated by LEC1. Regulatory sub-modules and functional regulatory elements implicated in seed development in soy were inferred by functional characterization of ChIP-Seq peaks at three different developmental stages. Moderate enrichment of the *CCAAT* matrix, composed of the core pentanucleotide with no obvious preference for flanking bases, was observed. We reanalyzed the complete collection of ChIP-Seq peaks using the same criteria that were applied to the analysis of Arabidopsis data, considering only intervals of 50 bps centered on peak summits. To limit confounding effects deriving from specific transcriptional patterns of the biological conditions under study, only peaks common to all the three developmental stages were retained. Overall, motif enrichment patterns recovered (Figure 4) are highly consistent with previous observations [33]. Notably, we observe substantial enrichment of *CCAAT* elements in global sites—second most enriched matrix—and in promoter—within 500 bps of an annotated TSS—regions (the fifth most enriched matrix). In the former, the *CCAAT* prevalence was robust, but not absolute, and single nucleotides were modestly enriched in the flanking nucleotides. In the latter, a *CCAA* sequence was more highly enriched than *CCAAT* (Figure 4b). Both matrices display a preferential *C* at position +1.

In summary, *in vivo* binding data for these sets of NF-Y HFD subunits are reassuringly consistent with the recognition of pentanucleotide *CCAAT* elements in plants, although we observe that flanking nucleotides are in general very variable with respect to the human matrix, and seem not to be consistent between different biological conditions.

## 3. Discussion

Recurrent expansion of TFs families in plants pose fundamental questions concerning the specificity of interactions and of DNA-binding. In the light of our knowledge of its biochemical features in mammals and yeast, NF-Y subunits represent an ideal model to dissect modes of interaction of expanded TF families in plants. There are at least two aspects for which the experiments presented here prove relevant: Similar efficiency of DNA-binding of two different AtNF-YAs and details of the DNA sequence-specificity of trimers.

That DNA-binding is a key aspect of NF-Y biology was illustrated by elegant genetic experiments of swapping the two external and the central HFD domains of the embryo-specific LEC1 and AtNF-YB3: The authors established that the embryo functions reside in the HFD of LEC1, and not in external domains [34]. Moreover, they pinpointed that residue Asp55 within the HFD is able to confer embryo functions to AtNF-YB3, a subunit involved in flowering. This aspartate is lysine in the mammalian NF-YB —and in the AtNF-YB2 used here— establishing non-sequence-specific DNA contacts. We ruled out the possibility of decreased, or indeed lack of DNA-binding by LEC1, and suggested an alternative explanation, involving repositioning of His50, another LEC1 diagnostic residue [35]. Thus, independently from the mechanistic details, it is clear that a substantial amount of regulation is embedded in DNA-binding contacts of the NF-Y trimer, including by the non-sequence-specific HFDs. In addition, several other TFs—LEC2, PIF1, bZIP67, TCL2—were shown to interact with LEC1, some recruiting it—likely with an NF-YC partner—to their specific binding sites [36]. DNA-recognition, as part of NF-Y/*CCAAT* or association with other TFs, are not mutually exclusive ways to impact on gene expression, and indeed the matrices, shown in Figure 4, likely reflect this combinatorial potential.

**Figure 5:**
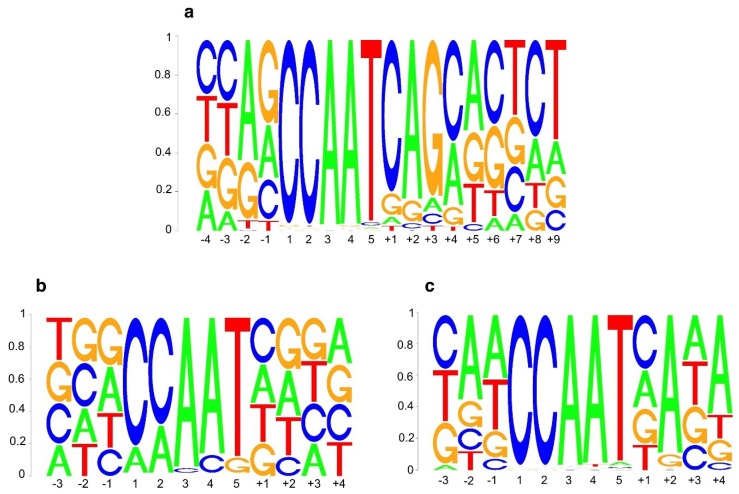
Comparison of sequence-specificity of *CCAAT* box elements in human and Arabidopsis NF-Y. (**a**) Human sequence logo of the *CCAAT* box element as derived from *de novo* motif discovery analysis of 12,655 NF-YB ChIP-Seq peaks from the human K562 cell line [3]. (**b**) *A. thaliana* sequence logo of the *CCAAT* box element as determined *in vitro* in this study by saturation mutagenesis of the AtNF-Y-bound *CAB2* promoter. See Figure 2. Note that *CCAAT* nucleotide bases were only analyzed with single base transversions. (**c**) *A. thaliana* sequence logo of the *CCAAT* box element as determined by *de novo* motif discovery analysis of 4563 overlapping ChIP-Seq peaks of AtNF-YB2 and AtNF-YC2. See also Figure 3. For consistency, in (a,b,c) base positions are numbered relative to the *CCAAT* pentanucleotide (positions 1−5 in each logo).

DNA-binding domains of sequence-specific TFs are typically the most conserved parts of these proteins. NF-Y is one of the most evolutionarily conserved family of TFs, and its “pioneering” role in chromatin opening and histone marks deposition has been proposed to be mediated by the overall histone-like structure. Not surprisingly, therefore, subunits interactions and DNA-binding subdomains of NF-Y homology regions are the most conserved in the three subunits. All animal kingdoms have one or, at most, two genes *per* NF-Y subunit, presenting a simple picture. Except for isoforms generated by alternative splicing events—which incidentally never involve DNA-binding domains—trimeric combinatorial possibilities are limited. In mammals, a highly specific and well-characterized DNA sequence motif —overall 10 bp— is centered on the *CCAAT* pentanucleotide—an almost absolute must—and on well-defined flanking sequences, as reported by numerous functional studies of promoter elements [9]. This motif is matched precisely by the verified affinity of NF-Y *in vitro* and its binding sites *in vivo,* revealed by several ChIP-Seq studies [3,21,22] (see Figure 5). In short, one DNA sequence, with little tolerated variation, is recognized by one trimer, with essentially no variation in its DNA-binding moieties. 

The radical expansion of the three NF-Y subunits gene families in plants theoretically brought several additional layers of complexity: In any given tissue/condition, hundreds of potential trimers could form in plants, provided that there would be no, or little, selectivity in heterodimer or heterotrimer formation. Y2H experiments indicated that plant NF-Y HFD subunits are generally capable of heterodimerization [37,38]. Structurally, the 3D structure of AtNF-YC3 in complex with the divergent NF-YB L1L (LEC1-LIKE 1; AtNF-YB6) support the hypothesis that HFD surfaces provide little selectivity [35]. Interactions were further detected in Y2H and Y3H experiments between single HFD subunits, or HFD heterodimers with NF-YAs [37,38,39]. For the time being, therefore, it is reasonable to assume the presence of a plethora of trimers in plants: The key issue then becomes their DNA-binding selectivity.

To measure affinities of different trimers for DNA, we reasoned that HFDs would partake marginally in selectivity for different sites, and thus, used two AtNF-YB2/AtNF-YC3 that are (i) the least divergent from the mammal ones, (ii) previously characterized in transcriptomics studies [18], (iii) involved in similar genetic pathways [13,15]. For one of these subunits, we also reanalyzed available ChIP-Seq data. We took a reductionistic approach by assaying the evolutionarily conserved domains of the subunits, which, unlike full-length proteins, are easy to produce and purify from soluble bacterial fractions: They retain *CCAAT* specificity. Note that the mammalian—and yeast—counterparts do the same, recapitulating very well the matrix bound *in vivo* by endogenous trimers based on ChIP-Seq experiments. We cannot formally rule out that external domains might influence DNA-binding, possibly not so much the strict sequence selectivity, but maybe other aspects, such as providing additional subunits-interaction contacts or modifying the bending angle: This has been previously shown, for the mammalian trimer [40,41]. In general, we felt that sequence preference could be dictated by the AtNF-YAs, in part because of our earlier observation using hybrid mammalian HFD and Arabidopsis NF-YA [37]. Because of the general conservation of the subunits-interaction and DNA-binding subdomains of plant NF-YAs, we used two members that show some differences, at least at the levels of primary sequences: The near-ubiquitous AtNF-YA2 and the AtNF-YA6 subunit which shows a more tissue-restricted pattern of expression. By and large, the two plant trimers showed similar affinity for *CCAAT*, which is a relevant observation: Indeed, even if these results will have to be confirmed by using multiple other configurations, for example with divergent HFD members (see below), our data suggest that the discriminating power of the individual trimers —and DNA affinity *per se*— might not be so relevant for the specific activity of the single NF-Y subunits in plants.

The results obtained to systematically derive the sequence preferred by a plant NF-Y *in vitro* are also important going forward. First, because they establish for the first time an actual plant *CCAAT* matrix, which includes positive data—oligos inhibiting binding—as well as negative ones, from oligos not competing. By comparing the mammal and plant matrices we can make two types of remark (see Figure 2b,c and Figure 5): (i) As far as the central *CCAAT* is concerned, mutations in any nucleotide is clearly detrimental for mammal and plant trimers, but the second *C* appears to be less important for the plant than for the mammal complex. (ii) With regard to the flanking nucleotides, the mammal trimer is, in general, far more selective, with mutations in certain positions being as dramatic as in the pentanucleotide. This observation does not apply to the plant NF-Y trimers tested in this study; common preferences emerge, for a purine at −1, for a *C* at +1, and a purine at +2. However, striking differences are also observed, the most important of which is strong selection by the mammal NF-Y against *T*s in essentially any position flanking *CCAAT*: A *T* at −1, as found in the *FT* and *CAB2 CCAAT*, would be extremely detrimental for mammal NF-Y binding *in vitro* [40], very rarely found in SELEX studies *in vitro* [42], and essentially never in ChIP-Seq data *in vivo* [3]. Additional differences are at positions +2 and +3, both very stringent for the mammalian complex, and much less so for plant NF-Y, although at the genomic level an *A* at +2 is significantly present in AtNF-YB2 and/or NF-YC3 sites. In summary, the mammal matrix has much higher information content than the plant one derived here, reflecting the higher sequence constraints.

Genome-wide analysis of Arabidopsis promoters revealed *CCAAT* as statistically enriched, but apparently devoid of the typical flankings found in animals [23]: This suggested that plant NF-Y complexes might have a reduced DNA specificity, strictly limited to the pentanucleotide. However, data provided here suggest an alternative explanation. First, our *in vitro* EMSAs suggest preferences for flanking nucleotides, as do ChIP-Seq data. Secondly, differences are observed between the flankings retrieved in AtNF-YB2 and/or NF-YC2, and soybean LEC1(NF-YB9) sites: These are unlikely to derive from species-specificity, given the similarity of soybean and Arabidopsis LEC1, as well as the similar timing of expression during embryo development. We, therefore, propose that higher flexibility of plant NF-Ys in DNA-recognition, specifically of nucleotides flanking *CCAAT* is accompanied by high variability of *CCAAT* sites in plant promoters. This might have to do about the requirement to accommodate TFs recognizing neighboring sequences, more than authentic selectivity of NF-Y trimers. As we have only tested two trimers, albeit, with divergent AtNF-YAs, further biochemical work needs to be done to extend this result to all possible trimers.

Promoters of genes downregulated in plant NF-YB and NF-YC mutants are enriched in elements resembling those recovered in ChIP-Seq experiments with TOC1/PRR proteins [15]. Here, as well as *CCAAT* motifs, together with a matrix similar to the *CCACA* element we previously described (see matrix 2, left panel of Figure 3a) [18], we recover a variety of elements that have previously been associated with DNA-binding by characterized TFs as enriched under HFD ChIP-Seq peaks. TOC1/PRR proteins contain CCT domains as do a number of *GATA*-binding domain-containing proteins, and the TIFFY and GATA domain-containing ZIM product, which were recently shown by DAP-Seq to bind the [*A/G*][*A/G*]*CCGT*[*T/C*] element that is enriched in several of the current analyses [43]. Accordingly, the enrichment of these elements in AtNF-YB and AtNF-YC peaks might be attributed to NF-CCT complexes, whose ultimate DNA-binding specificity could be defined by domains outside of the CCT itself. Consistent with this hypothesis, large scale Y2H data [23] recovered a single interaction between a CCT protein and an NF-YA subunit—BBX9 with AtNF-YA6—but multiple CCT/NF-YB and CCT/NF-YC interactions, including those of TOC1/PRR, ZIM and CCT/GATA factors with HFD components. On this note, recognition of diverse elements has also been reported for the LEC1 containing NF-Y HFD heterodimers, potentially driven by the interaction with other sequence-specific partners ([33]; reviewed in Reference [36]).

Taken together, the mutational, ChIP-Seq and available protein interactions data are compatible with a model of the increased flexibility of HFD subunits interactions in plants with respect to animals. It is likely that most, if not all, NF-YAs and CCTs mediate their activity through interactions with HFD subunits and Y2H data are consistent with higher-order complexes involving other TFs. These enrichments may result from a combination of direct and long distance chromatin interactions. This model would position HFD complexes as critical hubs for the integration of TF signaling networks involving diverse families of TFs regulating developmental, as well as stress—and light-related processes through chromatin modulation.

## 4. Materials and Methods 

### 4.1. Protein Expression, Purification 

Expression vectors for AtNF-YA6-6His (aa 170−237), AtNF-YA2-6His (aa 134−207) and HFD subunits vectors for co-expression of WT *A. thaliana* 6His-NF-YB2/NF-YC3 (AtNF-YB2 aa 24−116; AtNF-YC3 aa 55−148) were previously described [18,26]. Proteins were produced in *E. coli* and purified by nickel-ion metal affinity chromatography (IMAC) as described [26,37,44]. After protease cleavage with thrombin to remove the 6His-tag, At NF-YB2/NF-YC3 dimeric proteins were further purified by Gel Filtration chromatography (GF) in Buffer B (10 mM Tris-HCl pH 8.0, 400 mM NaCl, 2 mM DTT). 6His-tagged IMAC purified proteins used in EMSA were dialyzed against Buffer B containing 10% glycerol, frozen in liquid nitrogen and stored at −80 °C.

### 4.2. EMSAs and In Vitro Competition Analyses

For dose-response EMSAs, DNA binding reactions were assembled with GF purified HFD subunits (Figure S1), and IMAC purified AtNF-YA6, AtNF-YA2 in a binding mix containing the Cy5-labeled probe (20 nM) with the following final composition—12 mM Tris-HCl pH 8.0, 50 mM NaCl, 50 mM KCl, 5 mM MgCl2, 0.5 mM EDTA, 12% glycerol, 0.2 mg/mL BSA, 2.5 mM DTT, supplemented with 100 ng of poly(*dA:dT*). Binding reactions were incubated at 30 °C for 30’, and separated by electrophoresis on 6% polyacrylamide gels in 0.25X TBE. Prior to reaction assembly, serial dilutions of proteins were prepared in Dilution Buffer (Buffer B with an additional 10% glycerol and 0.1 mg/ml BSA). The Cy5-labeled 31 bp *CAB2 CCAAT* probe ([Cy5]*CTTAAAATCCAATGAATGAACAGATAAAGAT*) and unlabeled competitor oligo was derived from the *A. thaliana CAB2* (lhcb1*1) (AT1G29920) (−42 to −73 from TSS) promoter *CCAAT* box bound by the Tac complex [30]. The −5.3 kb *CCAAT* box *FT CCAAT* probe ([Cy5]*GCACTCATCCAATCCTTTATGGAATCTTCTT*) was previously described [25]. WT and mutant oligonucleotide sequences used in competition assays are listed in Table S1.

For competition EMSAs to define AtNF-Y sequence-specificity of Figure 2, thrombin cleaved GF purified At NF-YB2/NF-YC3 dimer was pre-mixed with AtNF-YA6 at 1:4.5 molar ratio, and incubated in binding mix with the labeled probe (20 nM) for 10’ at 30 °C, with a final concentration of 30 nM HFD dimer. Aliquots of the binding reaction were then supplemented with the unlabeled competitor oligo at increasing concentrations (20, 50 or 500 nM), or TE buffer, and further incubated at 30 °C for 30’ prior to electrophoresis.

Fluorescence gel images were acquired and analyzed with a ChemidocMP imaging system with the ImageLab Software (Bio-Rad Laboratories). Competitor oligo efficiency was calculated as follows: Percent bound probe was quantified in each data point of the dose curve, and plotted vs the competitor concentration (expressed as competitor/total oligo concentration). Competitor efficiency (slope/WT) represents the slope of the regression line obtained through the 0, 1x, 5x competition data points *vs* the WT oligo slope performed for each experiment. Data represent the mean of at least three independent competition experiments, +/− sd.

### 4.3. Analysis of AtNF-YB2, AtNF-YC2 and LEC1 Binding Sites In Vivo

Reference genomes and corresponding annotations for *A. thaliana* and *G. max* were retrieved from the Ensemble Plants genome browser [45]. Promoter elements were defined as genomic intervals from −500 bp to annotated TSSs. ChIP-Seq data, in the form of narrow peaks bed files, were retrieved from their respective repositories in the GEO database: GSE8056835 and GSE9988237. A custom Perl script was used to extract peak summits and to extend these regions by 25 bp upstream and downstream. The intersection of genomic coordinates of enriched ChIP-Seq peaks and promoter elements was performed using the BEDTools software [46]. Motifs enrichment analyses were performed on the extended summits using the Homer software package [32].

## Supplementary Materials

The following are available online at https://www.mdpi.com/2223-7747/8/10/406/s1, Figure S1: Recombinant proteins used in EMSAs, Table S1: Oligonucleotides.
