# Supplementary material for: The Plant NF-Y DNA Matrix In Vitro and In Vivo
Source: Plants (Basel). 2019 Oct 10;8(10):406. doi: 10.3390/plants8100406 (PMC6843132; doi:10.3390/plants8100406)
Supplement: Supplementary file 1 [file plants-08-00406-s001.pdf]

SUPPLEMENTARY DATA

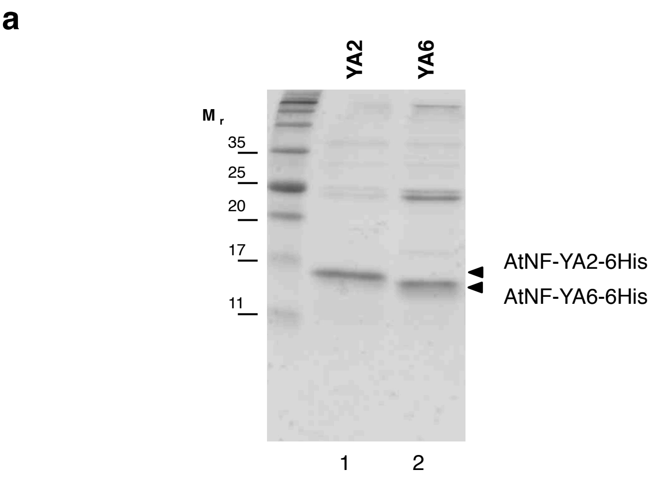

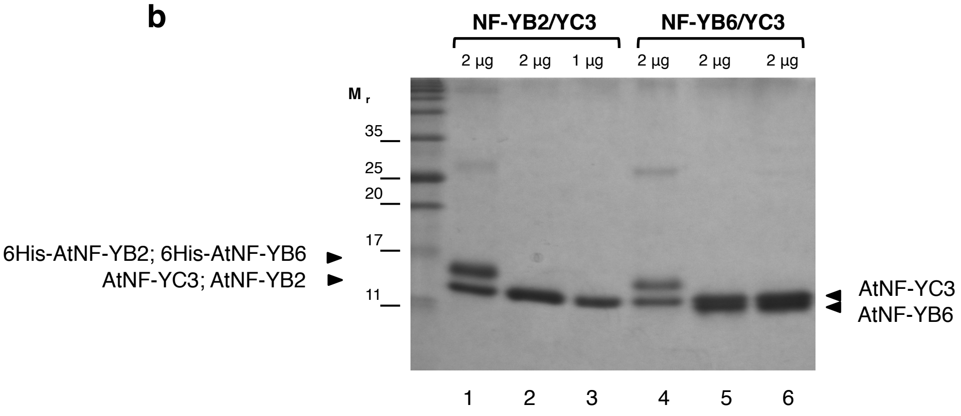

Figure S1. Recombinant proteins used in EMSAs.
SDS-PAGE gels were stained with Coomassie. Migration of relative molecular weight protein mass standards (Mr, expressed in kDa) is shown on the left of the gels.
(a) Lanes 1,2: 2 µg of IMAC purified 6His-tagged protein samples were loaded for At NF-YA2 and NF-YA6. (b) Lanes 1-3: At NF-YB2/NF-YC3 purified recombinant proteins, before (lane 1) and after (lanes 2, 3) 6His-tag removal by thrombin cleavage and GF purification, were loaded on SDS-PAGE gels. Amounts are indicated per each lane. Note that after proteolysis, cleaved AtNF-YB2 co-migrates with AtNF-YC3. Lanes 4-6: At NF-YB6/NF-YC3 IMAC purified recombinant protein samples, before (6His-tagged, lane 4) and after proteolysis and GF from different protein preparations (lanes 5, 6), used to obtain crystals of At NF-YB6/NF-YC3 [35] (PDB 5G49) were loaded as a reference for AtNF-Y subunits migration. Note that thrombin cleaved AtNF-YB6 (L1L) migrates faster than AtNF-YC3. Thrombin cleaved At NF-YB2/NFYC3 HFD dimers were used in EMSAs shown in this manuscript.

Table S1 - Oligonucleotides EMSA probes and competitor oligos used in this work*

Name	sequence (5'-3')	label
FT CCAAT	GCACTCATCCAATCCTTTATGGAATCTTCTT	5' Cy5
CAB2 CCAAT (-65)	CTTAAAATCCAATGAATGAACAGATAAAGAT	5' Cy5
CAB2 CCAAT (-65)/ WT	CTTAAAATCCAATGAATGAACAGATAAAGAT	na
$CAB2_{25}$	TTAAAATCCAATGAATGAACAGATA	na
CAB2 CCAAT (-245)	GCTACAACCCAATAACTAAAACTTAAAGTAT	na
Hsp70	CTTCTGAGCCAATCACCGAGCTCGATGAGGC	na
$Hsp70_{25}$	TTCTGAGCCAATCACCGAGCTCGAT	na
CAB2mut A5n	CTTAAcATCCAATGAATGAACAGATAAAGAT	na
	CTTAAgATCCAATGAATGAACAGATAAAGAT	na
	CTTAAtATCCAATGAATGAACAGATAAAGAT	na
CAB2mut A6n	CTTAAAcTCCAATGAATGAACAGATAAAGAT	na
	CTTAAAgTCCAATGAATGAACAGATAAAGAT	na
	CTTAAAtTCCAATGAATGAACAGATAAAGAT	na
CAB2mut T7n	CTTAAAAaCCAATGAATGAACAGATAAAGAT	na
	CTTAAAAcCCAATGAATGAACAGATAAAGAT	na
	CTTAAAAgCCAATGAATGAACAGATAAAGAT	na
CAB2mut C8A/ CAB2mut	CTTAAAATaCAATGAATGAACAGATAAAGAT	na
CAB2mut C9A	CTTAAAATCaAATGAATGAACAGATAAAGAT	na
CAB2mut A10C	CTTAAAATCCcATGAATGAACAGATAAAGAT	na
CAB2mut A11C	CTTAAAATCCAcTGAATGAACAGATAAAGAT	na
CAB2mut T12G	CTTAAAATCCAAgGAATGAACAGATAAAGAT	na
CAB2mut 11C12A	CTTAAAATCCAcaGAATGAACAGATAAAGAT	na
CAB2mut G13n	CTTAAAATCCAATaAATGAACAGATAAAGAT	na
	CTTAAAATCCAATcAATGAACAGATAAAGAT	na
	CTTAAAATCCAATtAATGAACAGATAAAGAT	na
CAB2mut A14n	CTTAAAATCCAATGcATGAACAGATAAAGAT	na
	CTTAAAATCCAATGgATGAACAGATAAAGAT	na
	CTTAAAATCCAATGtATGAACAGATAAAGAT	na
CAB2mut A15n	CTTAAAATCCAATGAcTGAACAGATAAAGAT	na
	CTTAAAATCCAATGAgTGAACAGATAAAGAT	na
	CTTAAAATCCAATGAtTGAACAGATAAAGAT	na
CAB2mut T16n	CTTAAAATCCAATGAAaGAACAGATAAAGAT	na
	CTTAAAATCCAATGAAcGAACAGATAAAGAT	na
	CTTAAAATCCAATGAAgGAACAGATAAAGAT	na

*mutant nucleotides are indicated in lowercase. Unlabeled complementary oligos were used for annealing dsDNA oligonucleotide competitors or probes. label: oligo modification; na: unmodified oligo
